# Pemafibrate ameliorates renal injury through induction of FGF21 and ketone body production in male mice

**DOI:** 10.14814/phy2.70135

**Published:** 2025-01-30

**Authors:** Kunihiko Takahara, Noriyuki Ouchi, Tomonobu Takikawa, Yuta Ozaki, Lixin Fang, Hiroshi Kawanishi, Minako Tatsumi, Yoshimitsu Yura, Katsuhiro Kato, Mikito Takefuji, Toyoaki Murohara, Koji Ohashi

**Affiliations:** ^1^ Department of Cardiology Nagoya University Graduate School of Medicine Nagoya Japan; ^2^ Department of Molecular Medicine and Cardiology Nagoya University Graduate School of Medicine Nagoya Japan

**Keywords:** FGF21, inflammation, pemafibrate, PPARα, β‐hydroxybutyrate

## Abstract

Chronic kidney disease is a life‐threatening disease worldwide. PPARα is a crucial transcriptional regulator of lipid metabolism and inflammation. Here, we examine whether a novel selective PPARα modulator, pemafibrate modulates renal injury in a model of unilateral ureteral obstruction (UUO). Administration of pemafibrate to wild‐type (WT) mice led to reduction of renal dysfunction and fibrosis after UUO with accompanying increases in plasma levels of fibroblast growth factor (FGF) 21 and ketone body β‐hydroxybutyrate (BHB). Treatment of WT mice with FGF21 or BHB precursor resulted in attenuation of renal fibrotic and inflammatory responses after UUO. Treatment of proximal tubular cells with FGF21 or BHB reduced expression of epithelial–mesenchymal transition markers. These findings suggest that pemafibrate could ameliorate renal damage, at least in part, by its abilities to increase the production of FGF21 and BHB.

## 1 INTRODUCTION

Chronic kidney disease (CKD) is among the most serious health problems, affecting approximately 850 million people worldwide (Jager et al., 2019; Kovesdy, 2022). It is associated with dyslipidemia, which is characterized by hypertriglyceridemia and low high‐density lipoprotein (HDL) cholesterol levels (Vaziri, 2014). CKD‐induced lipid disorders cause systemic inflammation, facilitating cardiovascular disease progression (Vaziri, 2014). Inflammation and metabolism, including fatty acid metabolism, pathways are dysregulated in diseased human kidneys (Kang et al., 2015).

Peroxisome proliferator‐activated receptor (PPAR)‐α is a key transcriptional regulator of lipid metabolism and inflammation (Brown & Plutzky, 2007; Staels et al., 1998). PPARα agonists are clinically used to decrease the plasma triglyceride levels and increase the HDL levels (Fruchart, 2009). PPARα agonists also regulate the cardiovascular function (Fruchart et al., 1999; Katayama et al., 2009; Okopien et al., 2017). PPARα plays important roles in preventing renal fibrosis and inflammation (Kang et al., 2015; Li et al., 2013). However, currently available PPARα agonists, such as fenofibrate and bezafibrate, increase the serum creatinine (Cr) levels (Catapano et al., 2016a, 2016b; Davidson et al., 2007). Pemafibrate is a novel selective PPARα modulator that exhibits higher PPARα agonistic activity and selectivity than all other PPARα agonists (Fruchart et al., 2021). Therefore, pemafibrate is potentially more effective in decreasing the triglyceride levels and inflammation than other existing PPARα agonists, thereby inhibiting dyslipidemia‐associated diseases, including cardiovascular and renal disorders (Hennuyer et al., 2016; Kawanishi et al., 2020). However, the specific effects of pemafibrate against renal diseases and the underlying molecular mechanisms remain unclear. Therefore, in this study, we aimed to investigate whether pemafibrate modulates renal injury using a unilateral ureteral obstruction (UUO) mouse model.

## 2 MATERIALS AND METHODS

### 2.1 Materials

Pemafibrate was provided by Kowa Co., Ltd. (Nagoya, Japan), and 1,3‐butanediol (BD) admixture diet was prepared using normal chow containing 20% 1,3‐BD solution (025‐07355; Wako). Recombinant human fibroblast growth factor (FGF)‐21 and human transforming growth factor (TGF)‐β1 proteins were purchased from R&D Systems (2539‐FG‐025 and 240‐B‐002, respectively). Plasma FGF21 levels were measured using an enzyme‐linked immunosorbent assay kit (MF2100; R&D Systems) (Kim et al., 2013). β‐hydroxybutyrate (BHB) was purchased from Sigma‐Aldrich (54920). BHB assay kit was purchased from Cayman (700190). Lipid profiles were analyzed using specific total cholesterol (635‐50981), HDL‐cholesterol (299‐96501), triglyceride (632‐50991) kits (FUJIFILM). Antibodies against vimentin (5741S), N‐cadherin (13116S), and α‐tubulin (2144S) were purchased from Cell Signaling Technology (Danvers, MA, USA). Antibodies against TGFβ1 (ab215715), collagen I (ab316222), and collagen III (ab184993) were purchased from Abcam. Monocyte/macrophage monoclonal antibody (MOMA)‐2 was purchased from Bio‐Rad (MCA519GT). Adenoviral vectors expressing full‐length mouse FGF21 (Ad‐FGF21) were constructed under the control of a CMV promoter, as previously described (Enomoto et al., 2011; Ouchi et al., 2008). Adenoviral vectors expressing β‐galactosidase (Ad‐β‐gal) were used as controls (Ouchi et al., 2004).

### 2.2 Animals and surgical procedure

Seven‐week‐old male wild‐type (WT) mice (Jackson Laboratory) were fed normal diets containing pemafibrate (0.12 mg/kg/day), 1,3‐BD, or vehicle for 2 weeks. At the age of 8 weeks, WT mice were subjected to UUO surgery under anesthesia. Anesthesia (medetomidine, midazolam, and butorphanol at 0.15, 2.0, and 2.5 mg/kg, respectively) was intraperitoneally administered, and its adequacy was confirmed by the lack of a toe‐pinch withdrawal response during the surgical procedure. Left ureter was ligated at two sites using the 5.0 silk suture. Ad‐β‐gal (3 × 10^8^ plaque‐forming units [PFU]) or Ad‐FGF21 (3 × 10^8^ PFU) was intravenously injected into the right jugular vein of mice 3 days prior to surgery, as previously described (Kawanishi et al., 2020). The study protocol was approved by the Institutional Animal Care and Use Committee of Nagoya University (approval number: M240087‐001). All animal experiments adhered to the relevant guidelines and regulations, including the Animal Research: Reporting of In Vivo Experiments guidelines.

### 2.3 Cell culture

HK‐2 cells were purchased from the American Type Culture Collection and cultured in the Dulbecco's modified Eagle's medium/F12 (Gibco) supplemented with 10% fetal bovine serum at intervals of 3–4 days. The cells were cultured in the Dulbecco's modified Eagle's medium/F12 for 16 h with or without pemafibrate, FGF21, and BHB for 1 h, followed by stimulation with TGFβ1 (10 ng/mL) or vehicle for 48 h. Then, 3‐hydroxy‐3‐methylglutaryl‐CoA synthase 2 (*HMGCS2*) was knocked‐down via cell transduction with 10 nM small interfering RNA (siRNA) using Lipofectamine RNAiMAX (Invitrogen) 24 h before the experiments. Lipofectamine RNAiMAX and siRNAs were dissolved in Opti‐MEM (Gibco). ON‐TARGETplus siRNA SMART pools targeting HMGCS2 were purchased from Horizon Discovery. Control cultures were transfected with an unrelated scrambled siRNA (ON‐TARGET plus Control Non‐Targeting Pool; Horizon Discovery).

### 2.4 Histology and immunohistochemistry

Tissue samples were fixed with 4% paraformaldehyde and embedded in paraffin. Serial tissue sections (5 μm) of the kidneys were stained with Masson's trichrome (Sigma‐Aldrich) and Picrosirius red (PSR‐1; ScyTek Laboratories, Inc.). Then, fibrotic area was measured using the Image J analysis system (Ohashi et al., 2007; Schneider et al., 2012). For immunohistochemistry, kidney sections were stained with MOMA‐2. Number of MOMA‐2‐positive cells was determined by calculating the number of stained macrophages in three images from random areas of interest in each tissue.

### 2.5 Laboratory tests

Seven days after operation, the mice were sacrificed and blood and urine samples were collected for analysis. Plasma concentrations of urea nitrogen (UN) and Cr were measured in a commercial laboratory (SRL Inc., Tokyo, Japan).

### 2.6 Quantification of mRNA levels

Gene expression levels were analyzed via quantitative real‐time polymerase chain reaction (PCR). RNAs from HK‐2 cells and kidney tissues were extracted using the RNeasy Mini Kit (74104; Qiagen) and reverse‐transcribed into cDNAs using the ReverTra Ace kit (FSQ‐201; TOYOBO). Real‐time PCR was performed using the Bio‐Rad real‐time PCR detection system with THUNDERBIRD SYBR qPCR mix (QPS‐101; TOYOBO) as a double‐standard DNA‐specific dye. All primers used in this study are listed in Table 1. Expression levels of transcripts were compared with those of 36B4 and normalized to the mean values of the controls.

**TABLE 1:** Primers used for quantitative RT‐PCR.

Mouse	
36B4:	Forward 5′‐GCTCCAAGCAGATGCAGCA‐3′
	Reverse 5′‐CCGGATGTGAGGCAGCAG‐3′
Collagen I:	Forward 5′‐GTCCCAACCCCCAAAGAC‐3′
	Reverse 5′‐CAGCTTCTGAGTTTGGTGATA‐3′
Collagen III:	Forward 5′‐TGGTTTCTTCTCACCCTTCTT‐3′
	Reverse 5′‐TGCATCCCAATTCATCTACGT‐3′
TGFβ1:	Forward 5′‐CACCGGAGAGCCCTGGATA ‐3′
	Reverse 5′‐TTCCAACCCAGGTCCTTCCT ‐3′
TNFα:	Forward 5′‐CGGAGTCCGGGCAGGT ‐3′
	Reverse 5′‐GCTGGGTAGAGAATGGATGAACA ‐3′
IL6:	Forward 5′‐GCTACCAAACTGGATATAATCAGG ‐3′
	Reverse 5′‐CCAGGTAGCTATGGTACTCCAGAA ‐3′
IL1β:	Forward 5′‐AGTTGACGGACCCCAAAAG ‐3′
	Reverse 5′‐AGCTGGATGCTCTCATCAGG ‐3′
FGF21:	Forward 5′‐GCTGCTGGAGGACGGTTACA‐3′
	Reverse 5′‐CACAGGTCCCCAGGATGTTG‐3′
HMGCS2:	Forward 5′‐AAACTTCGCTCACACCTGCT ‐3′
	Reverse 5′‐ACTTCCCTGCTTCCACATTG‐3′
PPARα:	Forward 5′‐TTGTGGCTGGTCAAGTTCGG‐3′
	Reverse 5′‐GCTCTCTGTGTCCACCATGT‐3′
Human	
36B4:	Forward 5′‐TGCTCAACATCTCCCCCTTCTC ‐3′
	Reverse 5′‐ACCAAATCCCATATCCTCGTCC ‐3′
N‐Cadherin:	Forward 5′‐TGTTTGACTATGAAGGCAGTGG‐3′
	Reverse 5′‐TCAGTCATCACCTCCACCAT‐1 3′
Vimentin:	Forward 5′‐TGCTTCTCTGGCACGTCTTG‐3′
	Reverse 5′‐GGACATGCTGTTCCTGAATCTG‐3′
PPARα:	Forward 5′‐CTGAAGCTGACAGCACTAC ‐3′
	Reverse 5′‐TGAGATTAGCCACCTACCC ‐3′
HMGCS2:	Forward 5′‐CAGCCATTCCCACACATGCTCA ‐3′
	Reverse 5′‐GACTTTATAAAGCCCCAAGACT ‐3′

Abbreviations: FGF21, fibroblast growth factor 21; HMGCS2, 3‐hydroxy‐3‐methylglutaryl‐CoA synthase 2; IL, interleukin; PPARα, peroxisome proliferator‐activated receptor α; TGFβ1, transforming growth factor β1; TNFα, tumor necrosis factor α.

### 2.7 Western blotting analysis

Kidney tissue samples were solubilized in a lysis buffer (9803S; Cell Signaling Technology) with a protease inhibitor cocktail (11697498001; Roche). Protein concentration was determined using the BCA protein assay kit (Thermo Scientific). Equal amounts of proteins were separated via denaturing sodium dodecyl sulfate‐polyacrylamide gel electrophoresis, transferred to polyvinylidene difluoride membranes (GE Healthcare), and incubated with primary antibodies, followed by incubation with horseradish peroxidase‐conjugated secondary antibodies. Protein signals were detected using the enhanced chemiluminescence primer system (GE Healthcare). Expression levels were determined by measuring the corresponding band intensities using the ImageJ software (National Institutes of Health) (Schneider et al., 2012). Relative intensity was evaluated by comparing with the α‐tubulin signal intensity.

### 2.8 Statistical analyses

Data are represented as the mean ± standard deviation. Differences in variables between two groups with a normal distribution were evaluated using an unpaired Student's *t*‐test. Differences among multiple groups were evaluated using one‐way analysis of variance, followed by Tukey's post hoc test. Differences in variables between two and among multiple groups with non‐normal distribution were analyzed using the Wilcoxon signed‐rank and Steel–Dwass tests, respectively. Data distribution was evaluated using the Shapiro–Wilk test. Statistical significance was set at *p* < 0.05. All statistical analyses were conducted using the JMP Pro 16 software (SAS).

## 3 RESULTS

### 3.1 Pemafibrate attenuates renal dysfunction and damage after UUO

**FIGURE 1:**
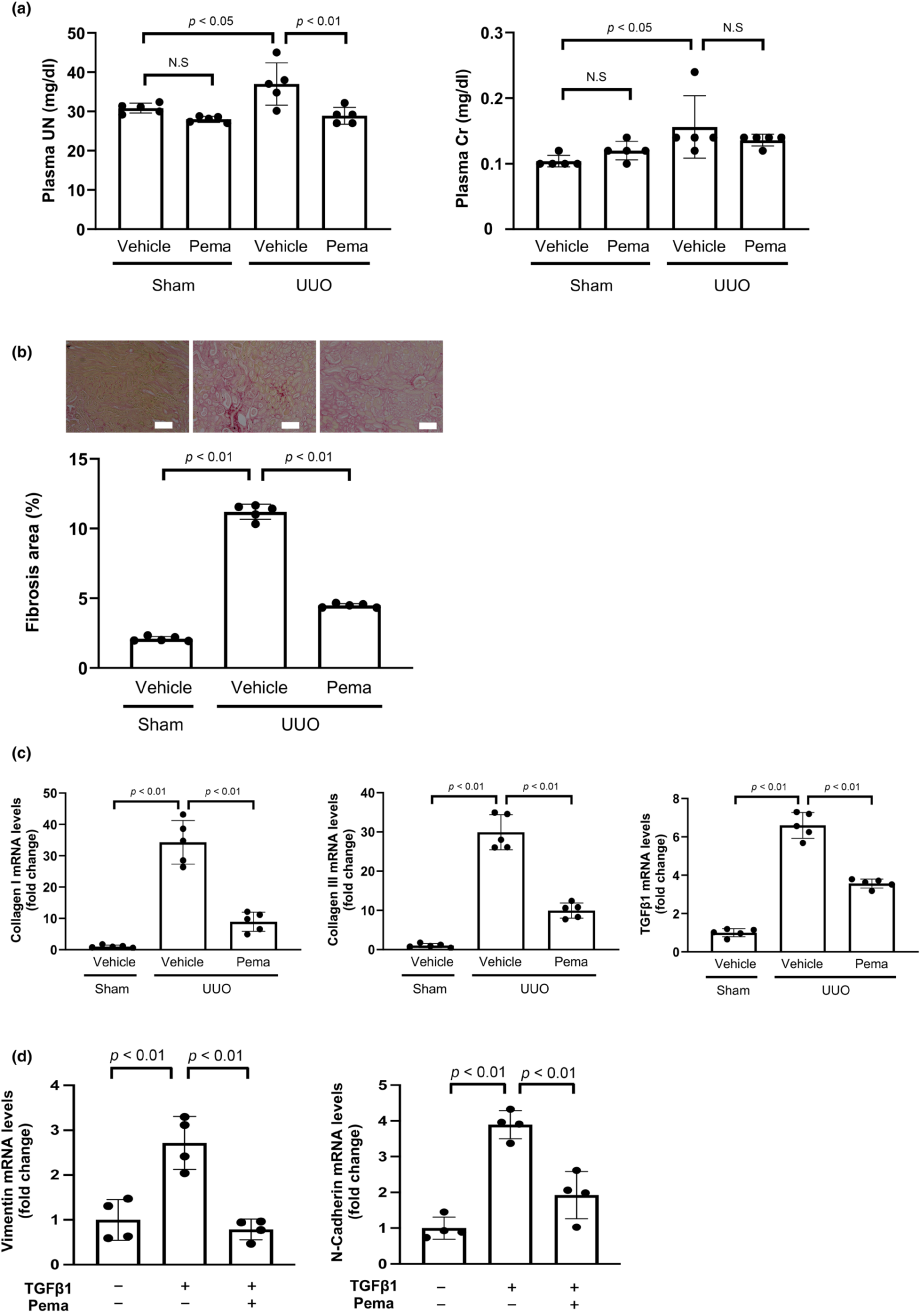
Pemafibrate (Pema) attenuates renal damage after unilateral ureteral obstruction (UUO). (a) Plasma parameters of renal function in wild‐type (WT) mice fed normal diets containing Pema or vehicle after UUO or sham operation. Left and right panels show the plasma concentrations of urea nitrogen (UN) and creatinine (Cr) in vehicle‐ and Pema‐treated WT mice after UUO and sham operations (*N* = 5/group). (b) Histological evaluation of tubulointerstitial fibrosis. Upper panels show the representative photographs of the kidneys of vehicle‐ and Pema‐treated mice after UUO and sham operations obtained after Picrosirius red staining. Lower panels show the quantitative analysis results of the fibrotic area determined using the ImageJ software (*N* = 5/group). Scale bars: 100 μm. (c) mRNA levels of fibrosis‐associated factors, such as collagen I, collagen III, and transforming growth factor (TGF)‐β1, in the kidneys of vehicle‐ and Pema‐treated mice after UUO and sham operations (*N* = 5/group). (d) mRNA levels of epithelial–mesenchymal transition (EMT) markers, such as N‐cadherin and vimentin, in HK‐2 cells. HK‐2 cells were pretreated with Pema (200 nM) or vehicle for 1 h, followed by incubation in the presence or absence of TGFβ1 (10 ng/mL) for 48 h (*N* = 4/group).

To examine the effects of pemafibrate on renal injury, WT mice were fed normal chow diets containing pemafibrate or vehicle, followed by UUO. Seven days after the UUO or sham operation, the mice were sacrificed for analysis after the collection of blood samples. UUO significantly increased the circulating levels of UN and Cr in control WT mice (Figure 1a). Pemafibrate treatment significantly reduced plasma UN levels in WT mice after UUO compared to vehicle treatment (Figure 1a). In contrast, plasma Cr levels after renal injury did not differ significantly between vehicle‐ and pemafibrate‐treated WT mice (Figure 1a). Pemafibrate treatment significantly reduced plasma triglyceride levels in both sham‐ and UUO‐operated WT mice (Table 2). In contrast, pemafibrate treatment significantly increased plasma total cholesterol and HDL cholesterol levels in both sham‐ and UUO‐operated WT mice (Table 2). UUO operation significantly reduced the expression of PPARα in the kidney (Figure S1a). Pemafibrate treatment did not affect the expression of PPARα in the kidney both after sham and UUO operations (Figure S1a).

**TABLE 2:** Lipid profiles of vehicle‐ and Pema‐treated WT mice after sham and UUO operation.

	Sham		UUO	
	Vehicle	Pema	Vehicle	Pema
Total cholesterol (mg/dl)	82.9 ± 3.2	123.3 ± 11.6*	90.5 ± 6.3	136.6 ± 1.8^#^
HDL cholesterol (mg/dl)	48.6 ± 3.4	62.7 ± 4.0*	56.0 ± 5.6	73.5 ± 4.5^#^
Triglyceride (mg/dl)	163.3 ± 2.6	123.3 ± 3.5*	146.8 ± 1.7	128.6 ± 0.9^#^

*Note*: Data are presented as mean ± SD.

* *p* < 0.01 for Sham/Vehicle group.

^#^ *p* < 0.01 for UUO/Vehicle group.

To evaluate tubulointerstitial fibrosis after UUO, the kidneys of WT mice after UUO or sham operation were stained with picrosirius red and Masson's trichrome. UUO increased the renal fibrotic area in WT mice, and pemafibrate treatment reduced the fibrotic area in the injured kidneys of WT mice after UUO compared with vehicle treatment (Figure 1b; Figure S1b). Treatment of WT mice with pemafibrate also decreased expression and protein levels of fibrosis markers, including collagen I, collagen III, and TGFβ1, in the injured kidney after UUO operation compared to vehicle treatment (Figure 1c; Figure S1c).

Epithelial–mesenchymal transition (EMT) of tubular epithelial cells is associated with kidney fibrosis (Hadpech & Thongboonkerd, 2023; Lovisa et al., 2015). To elucidate the mechanism of pemafibrate‐mediated attenuation of renal fibrosis in vivo, we examined the effect of pemafibrate on the expression of EMT markers in the human renal proximal tubular epithelial cell line, HK‐2. Pretreatment of HK‐2 cells with pemafibrate significantly decreased TGFβ1‐stimulated expression of EMT markers, including vimentin and N‐cadherin (Figure 1d). In consistent with pemafibrate treatment in in vivo, pemafibrate treatment did not affect the expression of PPARα in HK‐2 cells (Figure S1d).

### 3.2 FGF21 attenuates tubulointerstitial fibrosis after UUO

**FIGURE 2:**
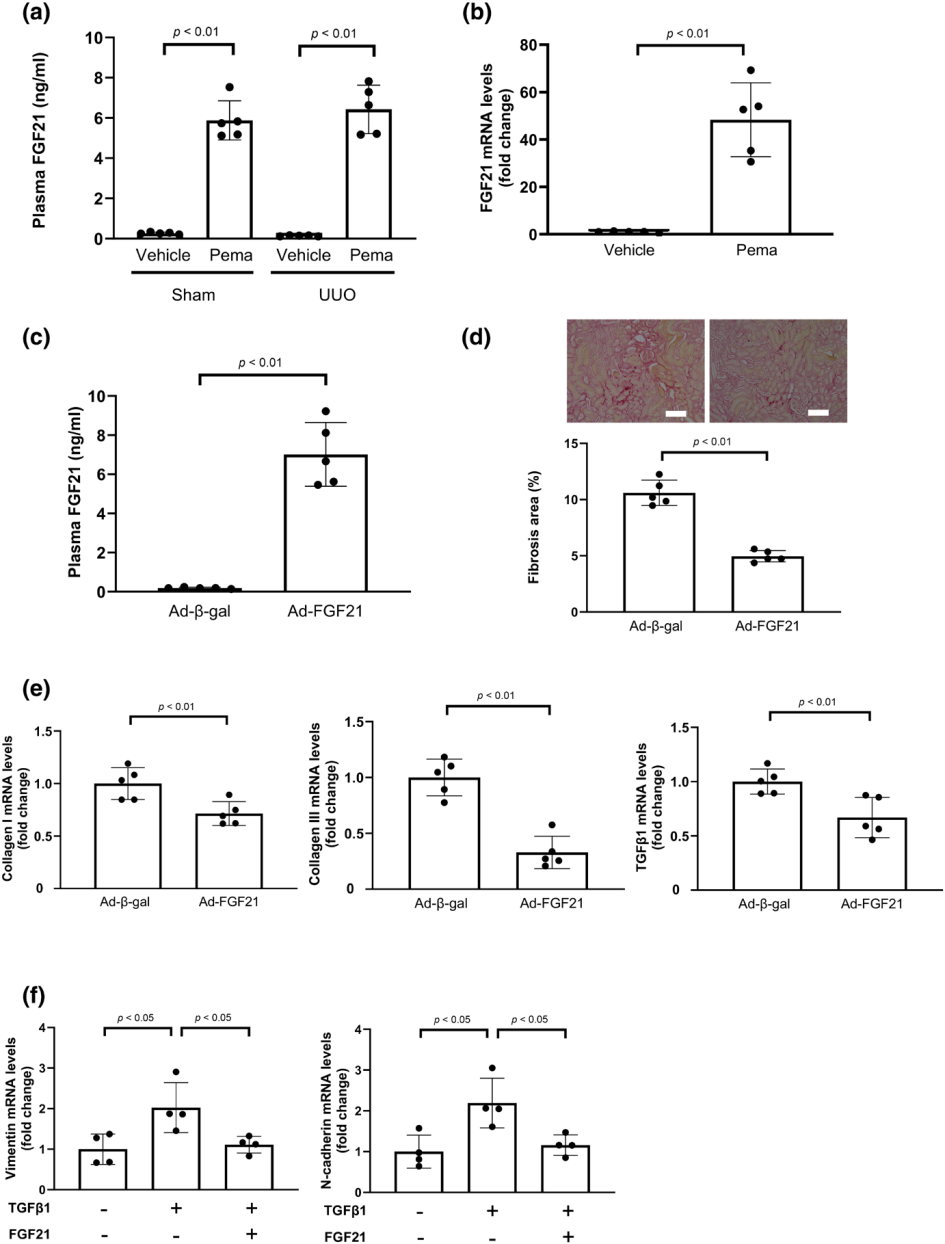
Role of fibroblast growth factor (FGF)‐21 in renal protection by Pema. (a) Plasma concentrations of FGF21 in vehicle‐ and Pema‐treated WT mice on day 7 after sham and UUO operations determined via enzyme‐linked immunosorbent assay (ELISA). (b) mRNA levels of *FGF21* in the liver on day 7 after UUO operation (*N* = 5/group). (c) Plasma concentrations of FGF21 in Ad‐FGF21‐ and control Ad‐β‐gal‐treated WT mice (N = 5/group). (d) Histological evaluation of tubulointerstitial fibrosis. Upper panels show the representative photos of the kidneys of Ad‐FGF21‐ and Ad‐β‐gal‐treated WT mice after UUO operation obtained after Picrosirius red staining. Lower panels show the quantitative analysis results of the fibrotic area determined using the Image J software (*N* = 5/group). Scale bars: 100 μm. (e) mRNA levels of fibrosis‐associated factors, such as collagen I, collagen III, and TGFβ1, in the kidneys of Ad‐β‐gal‐ and Ad‐FGF21‐treated mice after UUO operation (*N* = 5/group). (f) mRNA levels of EMT markers, such as N‐cadherin and vimentin, in HK‐2 cells. HK‐2 cells were pretreated with FGF21 protein (10 nM) or vehicle for 1 h, followed by incubation in the presence or absence of TGFβ1 (10 ng/mL) for 48 h (*N* = 4/group).

Because FGF21 is a target gene of PPARα (Lin et al., 2015), plasma concentration of FGF21 was measured in vehicle‐treated and pemafibrate‐treated WT mice. Treatment with pemafibrate robustly increased the circulating levels of FGF21 compared to the control on day 7 after UUO (Figure 2a). Because both PPARα and FGF21 are abundantly expressed in the liver (Goto et al., 2017; Nishimura et al., 2000), we assessed mRNA expression of FGF21 in the liver in vehicle‐treated and pemafibrate‐treated WT mice. Treatment of WT mice with pemafibrate significantly increased the mRNA levels of FGF21 in the liver, consistent with the results of a previous study (Kawanishi et al., 2020) (Figure 2b). Similarly, the treatment of WT mice with pemafibrate significantly increased the mRNA levels of FGF21 in the kidneys after UUO (Figure S2a).

Next, to evaluate whether FGF21 regulates renal function in vivo, adenoviral vectors expressing FGF21 (Ad‐FGF21) or control vector (Ad‐β‐gal) were intravenously injected into WT mice 3 days prior to surgery. Systemic administration of Ad‐FGF21 significantly increased plasma FGF21 concentration in WT mice at day 10 after adenoviral vector administration compared with Ad‐β‐gal treatment (Figure 2c). In addition, systemic administration of Ad‐FGF21 to WT mice significantly reduced renal fibrosis at day 7 after UUO operation compared with Ad‐β‐gal treatment (Figure 2d; Figure S2b). Consistently, Ad‐FGF21 treatment attenuated the mRNA and protein levels of fibrosis markers including collagen I, collagen III, and TGFβ1 in the injured kidney of WT mice after UUO operation (Figure 2e; Figure S2c). Furthermore, Ad‐FGF21 treatment reduced the protein levels of EMT markers including vimentin and N‐cadherin (Figure S2d). Consistently, pretreatment of HK‐2 cells with FGF21 significantly decreased TGFβ1‐stimulated expression of EMT markers including vimentin and N‐cadherin (Figure 2f).

### 3.3 Ketone body precursor attenuates tubulointerstitial fibrosis after UUO

**FIGURE 3:**
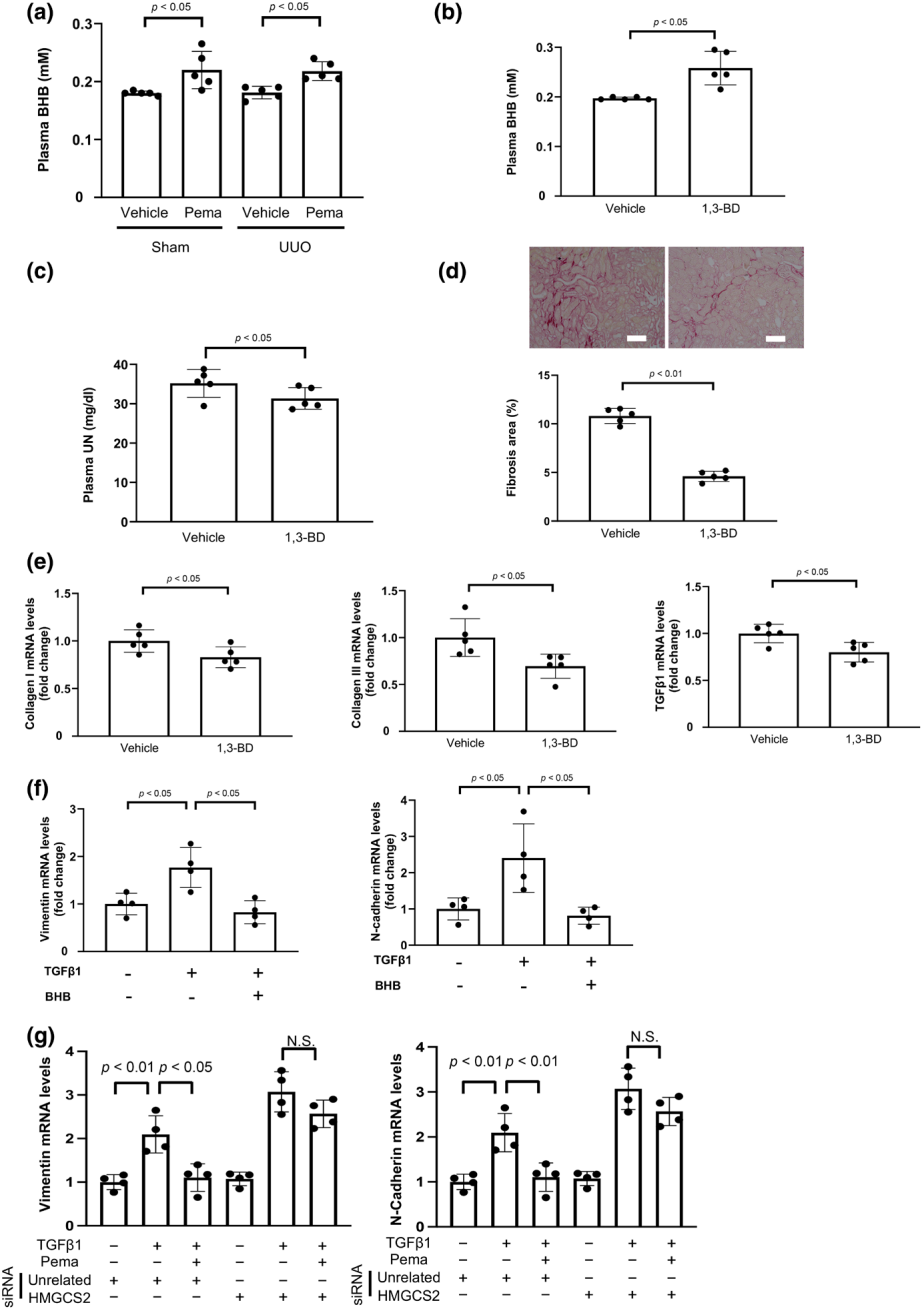
Role of β‐hydroxybutyrate (BHB) in renal protection by Pema. (a) Plasma BHB levels in vehicle‐ and Pema‐treated WT mice on day 7 after UUO operation (*N* = 5/group). (b) Plasma BHB levels in 1,3‐butanediol (BD)‐ and vehicle‐treated WT mice after UUO operation (*N* = 5/group). (c) Plasma UN levels in vehicle‐ and 1,3‐BD‐treated WT mice after UUO operation (*N* = 5/group). (d) Histological evaluation of tubulointerstitial fibrosis. Upper panels show the representative photographs of the kidneys of vehicle‐ and 1,3‐BD‐treated WT mice after UUO operation obtained after Picrosirius red staining. Lower panels show the quantitative analysis results of fibrotic area determined using the Image J software (*N* = 5/group). Scale bars: 100 μm. (e) mRNA levels of fibrosis‐associated factors, such as collagen I, collagen III, and TGFβ1, in the kidneys of 1,3‐BD‐ and vehicle‐treated mice after UUO operation (*N* = 5/group). (f) mRNA levels of EMT markers, such as N‐cadherin and vimentin, in HK‐2 cells. HK‐2 cells were pretreated with BHB (20 mM) or vehicle for 1 h, followed by incubation in the presence or absence of TGFβ1 (10 ng/mL) for 48 h (*N* = 4/group). (g) Role of 3‐hydroxy‐3‐methylglutaryl‐CoA synthase 2 (HMGCS2) in the inhibitory effect of Pema on TGFβ1‐stimulated expression of EMT markers, including vimentin and N‐cadherin, in HK‐2 cells. HK‐2 cells were treated with a small interfering RNA (siRNA) targeting HMGCS2 (10 nmol/L) or control unrelated siRNA for 8 h. After 16 h of serum starvation, HK‐2 cells were treated with Pema (200 nM) or vehicle for 1 h, followed by stimulation with TGFβ1 (10 ng/mL) or vehicle for 48 h (*N* = 4/group).

PPARα is one of the key regulators for ketone body synthesis, and ketone bodies exert beneficial effects on kidney disease (Fang et al., 2023; Grabacka et al., 2016; Hattori, 2021; Tomita et al., 2020). Thus, we evaluated plasma levels of the ketone body, BHB, after UUO surgery. Treatment of WT mice with pemafibrate increased plasma BHB levels after both sham and UUO operations compared to vehicle treatment (Figure 3a). Similarly, the treatment of WT mice with pemafibrate significantly increased BHB levels in the kidneys after both sham and UUO (Figure S3a). Since ketone bodies are synthesized in the liver (Grabacka et al., 2016), we assessed the expression of HMGCS2, a key enzyme in the livers of vehicle‐ and pemafibrate‐treated WT mice. Administration of pemafibrate to WT mice increased HMGCS2 expression in the liver compared to vehicle treatment (Figure S3b).

To evaluate the effects of BHB on renal injury, WT mice were fed normal chow diets containing 1,3‐BD, a precursor of BHB, or vehicle, followed by UUO. Seven days after UUO, the mice were sacrificed for analysis after collection of blood samples. Treatment with the 1,3‐BD admixture diet increased BHB concentration in the plasma of WT mice on day 7 after UUO compared to vehicle treatment (Figure 3b). Treatment with the 1,3‐BD admixture diet significantly reduced plasma UN levels and renal fibrosis in WT mice on day 7 after UUO compared to vehicle treatment (Figure 3c,d; Figure S3c). Consistently, treatment with 1,3‐BD admixture diet attenuated the mRNA and protein levels of fibrosis markers including collagen I, collagen III, and TGFβ1 in the injured kidney in WT mice after UUO operation (Figure 3e; Figure S3d). Treatment with the 1,3‐BD admixture diet also reduced the protein levels of EMT markers, including vimentin and N‐cadherin (Figure S3e). Pretreatment of HK‐2 cells with BHB significantly decreased TGFβ1‐stimulated expression of EMT markers including vimentin and N‐cadherin (Figure 3f). To assess the precise mechanism of the pemafibrate‐mediated reduction in EMT, HK‐2 cells were transfected with siRNAs targeting HMGCS2 or unrelated siRNAs. Transduction of HK‐2 cells with siRNA for HMGCS2 decreased mRNA levels of HMGCS2 by 83.7 ± 1.5% (*p* < 0.01). Knockdown of HMGCS2 blocked the inhibitory effect of pemafibrate on TGFβ1‐stimulated expression of EMT markers including vimentin and N‐cadherin (Figure 3g). Thus, pemafibrate inhibits EMT via HMGCS2‐mediated production of BHB, thereby attenuating renal fibrosis by protecting the kidneys.

### 3.4 Pemafibrate attenuates the renal inflammatory responses after UUO

**FIGURE 4:**
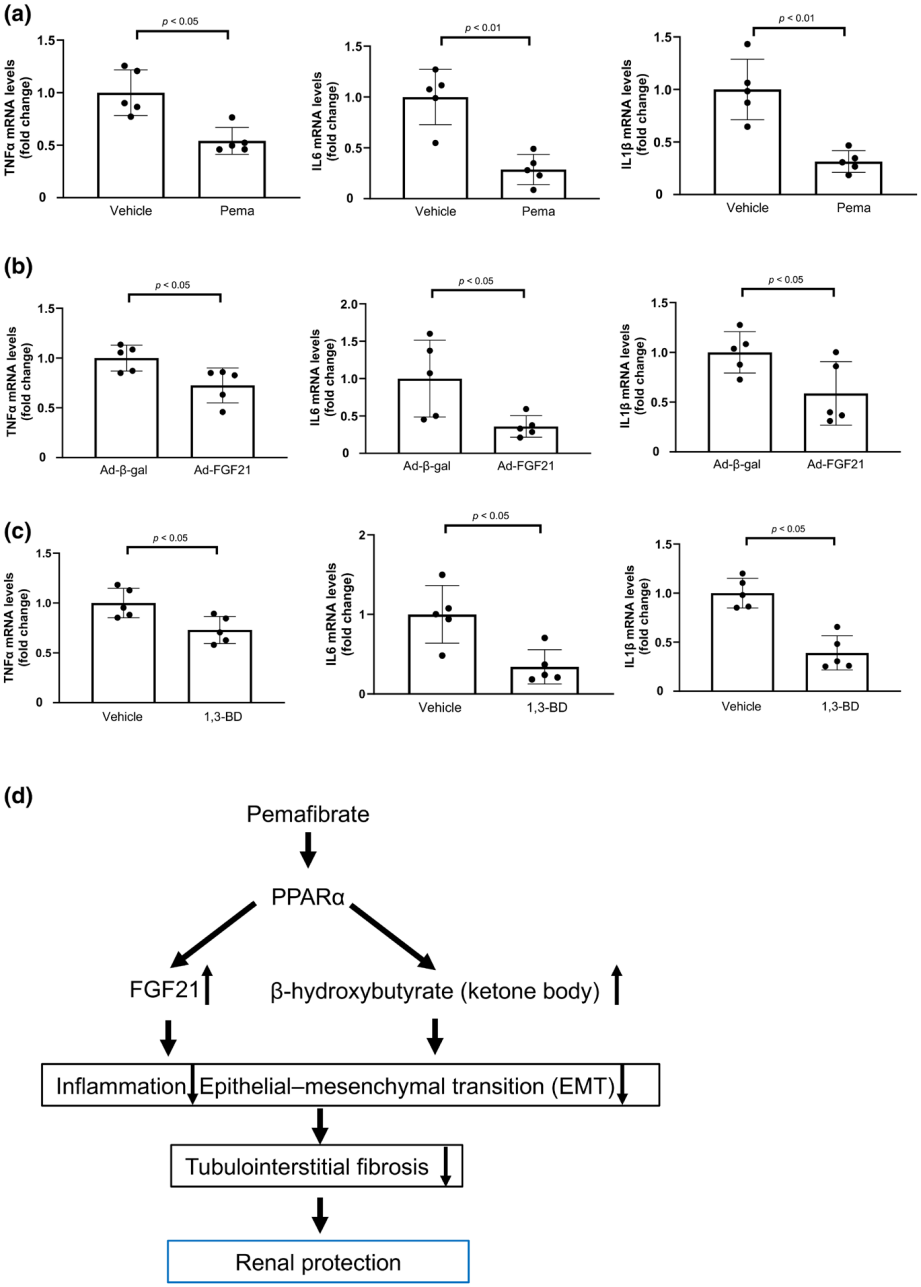
Systemic administration of Pema, FGF21, and BHB precursor attenuates the renal inflammatory responses after UUO operation. (a) mRNA levels of inflammatory mediators, such as the tumor necrosis factor (TNF)‐α, interleukin (IL)‐6, and IL1β, in the injured kidneys of Pema‐ and vehicle‐treated WT mice after UUO operation (*N* = 5/group). (b) mRNA levels of inflammatory mediators, such as TNFα, IL6, and IL1β, in the injured kidneys of Ad‐β‐gal‐ and Ad‐FGF21‐treated WT mice after UUO operation (*N* = 5/group). (c) mRNA levels of inflammatory mediators, such as TNFα, IL6, and IL1β, in the injured kidneys of 1,3‐BD‐ and vehicle‐treated WT mice after UUO operation (*N* = 5/group). (d) Proposed mechanism of renal protection by Pema. Pema attenuated tubulointerstitial fibrosis by inhibiting renal inflammation and EMT partly by increasing the production of FGF21 and ketone bodies, thereby protecting the kidneys.

Chronic inflammation contributes to the fibrotic process in kidney injury (Meng et al., 2014). Thus, to investigate whether pemafibrate modulates the inflammatory response in injured kidneys, the mRNA levels of inflammatory mediators in the kidneys of vehicle‐treated and pemafibrate‐treated WT mice were measured using quantitative real‐time PCR. Pemafibrate treatment reduced mRNA levels of pro‐inflammatory mediators, including tumor necrosis factor (TNF)‐α, interleukin (IL)‐6, and IL1β in the injured kidneys of WT mice compared with vehicle treatment (Figure 4a). In addition, systemic administration of Ad‐FGF21 to WT mice reduced the mRNA levels of TNFα, IL6, and IL1β in the injured kidney after UUO surgery (Figure 4b). Furthermore, treatment of WT mice with 1,3‐BD admixture diet reduced the mRNA levels of TNFα, IL6, and IL1β in the injured kidney after UUO surgery (Figure 4c).

The renal infiltration of immune cells, including macrophages, is a crucial pathological step in chronic inflammation that leads to tubulointerstitial fibrosis and renal injury (Cao et al., 2015). Thus, we evaluated the infiltration of macrophages into injured kidneys using immunohistochemistry for MOMA‐2. Pemafibrate treatment reduced the number of MOMA‐2‐positive macrophages in the injured kidneys of WT mice compared to vehicle treatment (Figure S4a). Systemic administration of Ad‐FGF21 to WT mice reduced the number of macrophages in injured kidneys after UUO surgery (Figure S4b). Furthermore, treatment of WT mice with a 1,3‐BD admixture diet reduced the number of macrophages in injured kidneys after UUO surgery (Figure S4c).

Collectively, pemafibrate administration may reduce inflammatory responses in injured kidneys, at least in part, by increasing the production of FGF21 and BHB.

## 4 DISCUSSION

This study demonstrated that pemafibrate, a novel selective PPARα modulator, alleviates renal damage in vivo. Systemic administration of pemafibrate attenuates blood UN levels and renal fibrosis in a UUO mouse model. Pemafibrate administration also increased plasma levels of FGF21 and BHB in UUO mice. Increased plasma levels of FGF21 following intravenous administration of Ad‐FGF21 led to a reduction in renal fibrosis in mice after UUO. Increased plasma levels of BHB following the oral administration of the ketogenic ingredient also led to reduced renal fibrosis in mice after UUO. In addition, treatment of cultured proximal tubular cells with FGF21 or BHB resulted in attenuated EMT responses to TGFβ1. Furthermore, the systemic administration of pemafibrate, FGF21, or a ketogenic ingredient attenuated renal inflammatory responses in UUO mice. These findings indicate that pemafibrate protects against renal dysfunction and fibrosis by reducing inflammatory and EMT responses and increasing circulating levels of FGF21 and BHB.

EMT of tubular epithelial cells contributes to the development of renal fibrosis and CKD progression in animal models (Holian et al., 2008; Lan, 2003; Lovisa et al., 2015; Shimizu et al., 2006). TGFβ1 is the most representative inducer of EMT in many fibrotic diseases including liver cirrhosis, myocardial infarction, and CKD (Pardali et al., 2017; Xu et al., 2009). TGFβ1 promotes renal fibrosis during CKD progression partly by inducing EMT of proximal tubular cells (Liu, 2004). In the present study, treatment of cultured renal proximal tubular cells with FGF21 and BHB reduced TGFβ1‐stimulated expression of vimentin and N‐cadherin, which are representative EMT markers. Thus, it is conceivable that pemafibrate attenuates tubulointerstitial fibrosis by reducing EMT of tubular cells through its ability to increase FGF21 and BHB production (Figure 4d).

Prolonged or excessive inflammation of the kidney is believed to cause renal fibrosis and contribute to the development of CKD. Circulating levels of inflammatory mediators, such as TNFα and IL6, are higher in patients with CKD than in normal subjects (de Vinuesa et al., 2006; Upadhyay et al., 2011). The current study demonstrated that pemafibrate reduced the expression of TNFα and IL6 in the injured kidneys. In addition, systemic administration of FGF21 or BHB reduced the expression of TNFα and IL6 in injured kidneys. These data suggest that pemafibrate exerts a renoprotective function by inhibiting the renal inflammatory response through FGF21‐ and BHB‐dependent mechanisms (Figure 4d).

FGF21 belongs to the superfamily of FGFs, which have various biological functions such as tissue development, regeneration, and metabolism. FGF21 plays an important role in regulating lipid metabolism and cardiovascular function (Degirolamo et al., 2016; Joki et al., 2015). FGF21 exerts renoprotective effects against cisplatin‐induced acute kidney injury and salt‐sensitive hypertension‐induced nephropathy (Chen et al., 2020; Li et al., 2018; Weng et al., 2021). In addition, FGF21 exerts antifibrotic effects on the liver and lung (Kang et al., 2018; Xu et al., 2016; Zhang et al., 2018). Furthermore, FGF21 overexpression via intravenous plasmid injection attenuated UUO‐induced renal fibrosis and inflammatory responses in mice (Zhong et al., 2024). Consistent with these observations, our data showed that the systemic administration of FGF21 reduced renal fibrotic and inflammatory responses to UUO. These data suggest that pemafibrate ameliorates CKD development, at least in part, through FGF21‐mediated renoprotective effects.

Ketone bodies, including BHB, acts as simple alternative energy sources under starvation. Additionally, ketone bodies, especially BHB, act as crucial regulators of systemic organ homeostasis, including renal homeostasis (Rojas‐Morales et al., 2020). Treatment with 1,3‐butandiol, which is a precursor of BHB, ameliorated salt‐induced hypertension and renal dysfunction in salt‐sensitive hypertensive rats and chronic kidney injury in a 5/6 nephrectomized mouse model (Ishimwe et al., 2020; Tomita et al., 2020). In agreement with these findings, the present study demonstrates that treatment with 1,3‐butandiol improved UUO‐induced renal fibrosis in mice. PPARα is a transcriptional activator of HMGCS2 which regulates ketone body production in the liver (Rodriguez et al., 1994). We recently reported that the anti‐inflammatory adipokine, adipolin, protects against renal injury by increasing HMGCS2‐dependent BHB production through PPARα activation (Fang et al., 2023). In this study, PPARα activation by pemafibrate led to enhanced HMGCS2 expression in the liver with accompanying increases in plasma BHB levels. Collectively, these observations suggest that PPARα activation by pemafibrate could promote HMGCS2‐inducible BHB production, thereby contributing to protection against renal injury.

To the best of our knowledge, this study is the first to demonstrate the protective effects of pemafibrate, a novel selective PPARα modulator, against UUO‐induced CKD by attenuating the inflammatory and EMT responses via FGF21‐ and BHB‐dependent mechanisms (Figure 4d). Collectively, our findings suggest PPARα as a potential therapeutic target for kidney diseases.

## FUNDING INFORMATION

This work was supported by Grant‐in‐Aid for Scientific Research A (2624H00677) and grants from Takeda Science Foundation (2600007051) to N Ouchi. K. Ohashi was supported by Grant‐in‐Aid for Scientific Research C (2624K10571).

## CONFLICT OF INTEREST STATEMENT

The authors have declared that no conflict of interest exists.

## ETHICS STATEMENT

The study protocol was approved by the Institutional Animal Care and Use Committee of Nagoya University (approval number: M240087‐001). All animal experiments adhered to the relevant guidelines and regulations, including the Animal Research: Reporting of In Vivo Experiments guidelines.

## Supporting information


Figures S1–S4.

## Data Availability

All the data are included in the main text. Any additional information of data can be available from the corresponding authors on request.
