# Supplementary material for: Pemafibrate ameliorates renal injury through induction of FGF21 and ketone body production in male mice
Source: Physiol Rep. 2025 Jan 30;13(3):e70135. doi: 10.14814/phy2.70135 (PMC11780494; doi:10.14814/phy2.70135)

Supplemental Figure Legends

Figure S1. Pemafibrate administration reduces renal fibrosis after UUO operation.

(A) mRNA levels of PPARα in the kidney from vehicle-treated and pemafibrate (Pema)-treated WT mice after UUO or sham operation (N = 5/group). **(B)** Histological evaluation of tubulointerstitial fibrosis. Upper panels show representative photos of the kidneys from vehicle-treated and pemafibrate (Pema)-treated mice after UUO or sham operation as determined by Masson Trichrome staining. Lower panel shows quantitative analysis of fibrosis area as measured by Image J (N = 5/group). Scale bars: 100 μm. **(C)** Protein levels of fibrosis-associated factors such as collagen I, collagen III and transforming growth factor (TGF) β1 in the kidney from vehicle-treated and pemafibrate (Pema)-treated mice after UUO or sham operation. Left panels show representative Western blots of Collagen I, Collagen III, TGFβ1 and Tubulin. Right panels show quantitative analyses of Collagen I/Tubulin, Collagen III/Tubulin and TGFβ1/Tubulin (N = 4/group). **(D)** mRNA levels of PPARα in HK-2 cells. HK-2 cells were treated with pemafibrate (200 nM) or vehicle for 3 h (N = 4/group).

Figure S2. Systemic administration of FGF21 reduces renal fibrosis and epithelial mesenchymal transition (EMT) after UUO operation. (A) mRNA levels of FGF21 in the kidney from vehicle-treated and pemafibrate (Pema)-treated mice after UUO operation (N = 5/group). **(B)** Histological evaluation of tubulointerstitial fibrosis. Upper panels show representative photos of the kidneys from Ad-FGF21-treated or Ad-β-gal-treated WT mice after UUO operation as determined by Masson Trichrome staining. Lower panel shows quantitative analysis of fibrosis area as measured by Image J (N = 5/group). Scale bars: 100 μm. **(C)** Protein levels of fibrosis-associated factors such as collagen I, collagen III and transforming growth factor (TGF) β1 in the kidney from Ad-FGF21-treated or Ad-β-gal-treated WT mice after UUO operation. Left panels show representative Western blots of Collagen I, Collagen III, TGFβ1 and Tubulin. Right panels show quantitative analyses of Collagen I/Tubulin, Collagen III/Tubulin and TGFβ1/Tubulin (N = 4/group). **(D)** Protein levels of EMT markers such as N-cadherin and vimentin in the kidney from Ad-FGF21-treated or Ad-β-gal-treated WT mice after UUO operation. Left panels show representative Western blots of vimentin, N-cadherin and Tubulin. Right panels show quantitative analyses of Vimentin/Tubulin and N-

Cadherin/Tubulin (N = 5/group).

Figure S3. Systemic administration of 1,3-butanediol (BD) reduces renal fibrosis and epithelial mesenchymal transition (EMT) after UUO operation. (A) Renal BHB levels of vehicle-treated or pemafibrate (Pema)-treated WT mice at day 7 after UUO operation (N = 5/group). **(B)** mRNA levels of 3-hydroxy-3-methylglutaryl-CoA synthase (HMGCS) 2, a key enzyme producing BHB, in the liver in vehicle-treated or pemafibrate (Pema)-treated WT mice after UUO operation (N = 5/group). **(C)** Histological evaluation of tubulointerstitial fibrosis. Upper panels show representative photos of the kidneys from vehicle-treated and 1,3-butanediol (BD)- treated WT mice after UUO operation as determined by Masson Trichrome staining. Lower panel shows quantitative analysis of fibrosis area as measured by Image J (N = 5/group). Scale bars: 100 μm. **(D)** Protein levels of fibrosis-associated factors such as collagen I, collagen III and transforming growth factor (TGF) β1 in the kidney from vehicle-treated and 1,3-BD-treated WT mice after UUO operation. Left panels show representative Western blots of Collagen I, Collagen III, TGFβ1 and Tubulin. Right panels show quantitative analyses of Collagen

I/Tubulin, Collagen III/Tubulin and TGFβ1/Tubulin (N = 4/group). **(E)** Protein levels of EMT markers such as N-cadherin and vimentin in the kidney from vehicle-treated and 1,3-BD-treated WT mice after UUO operation. Left panels show representative Western blots of vimentin, N-cadherin and Tubulin. Right panels show quantitative analyses of Vimentin/Tubulin and N-Cadherin/Tubulin (N = 4/group).

Figure S4. Systemic administration of pemafibrate, FGF21 or BHB precursor reduces infiltration of macrophages into the injured kidney after UUO operation. (A,B and C) Histological analysis of macrophages infiltration to the injured kidney after UUO operation as determined by monocyte/macrophage (MOMA) 2 staining. Upper panels show representative images of immuno-histochemical staining for MOMA2 from vehicle-treated and pemafibrate (Pema)-treated wild-type (WT) mice **(A)**, from Ad-FGF21-treated or Ad-β-gal-treated WT mice **(B)** and from vehicle-treated and 1,3-BD-treated WT mice **(C)** after UUO operation. Lower panels show quantitative analysis of MOMA2-positive cells in the kidney from vehicle-treated and Pema-treated WT mice **(A)**, from Ad-FGF21-treated or Ad-β-gal-treated WT mice **(B)** and from vehicle-treated and

1,3-BD-treated WT mice **(C)** after UUO operation (N = 5/group). Scale bars: 100 μm.

Figure S1

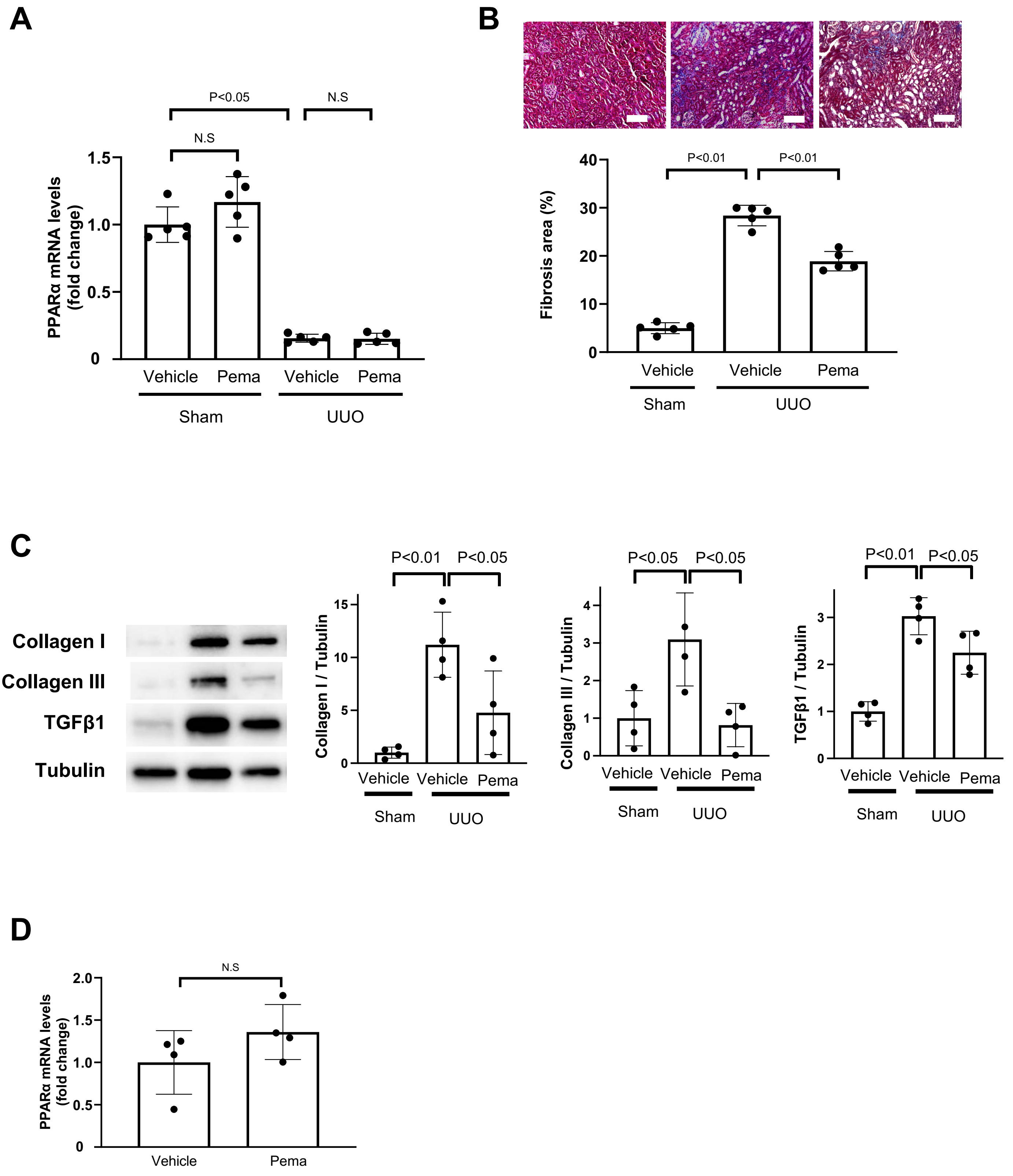

A
P<0.05
N.S
N.S
PPARα mRNA levels (fold change)
1.5
1.0
0.5
0
Vehicle
Pema
Vehicle
Pema
Sham
UUO
B
Fibrosis area (%)
P<0.01
P<0.01
40
30
20
10
0
Vehicle
Vehicle
Pema
Sham
UUO
C
Collagen I
Collagen III
TGFβ1
Tubulin
P<0.01
P<0.05
Collagen I / Tubulin
15
10
5
0
Vehicle
Vehicle
Pema
Sham
UUO
P<0.05
P<0.05
Collagen III / Tubulin
4
3
2
1
0
Vehicle
Vehicle
Pema
Sham
UUO
P<0.01
P<0.05
TGFβ1 / Tubulin
3
2
1
0
Vehicle
Vehicle
Pema
Sham
UUO
D
N.S
PPARα mRNA levels (fold change)
2.0
1.5
1.0
0.5
0
Vehicle
Pema

Figure S2

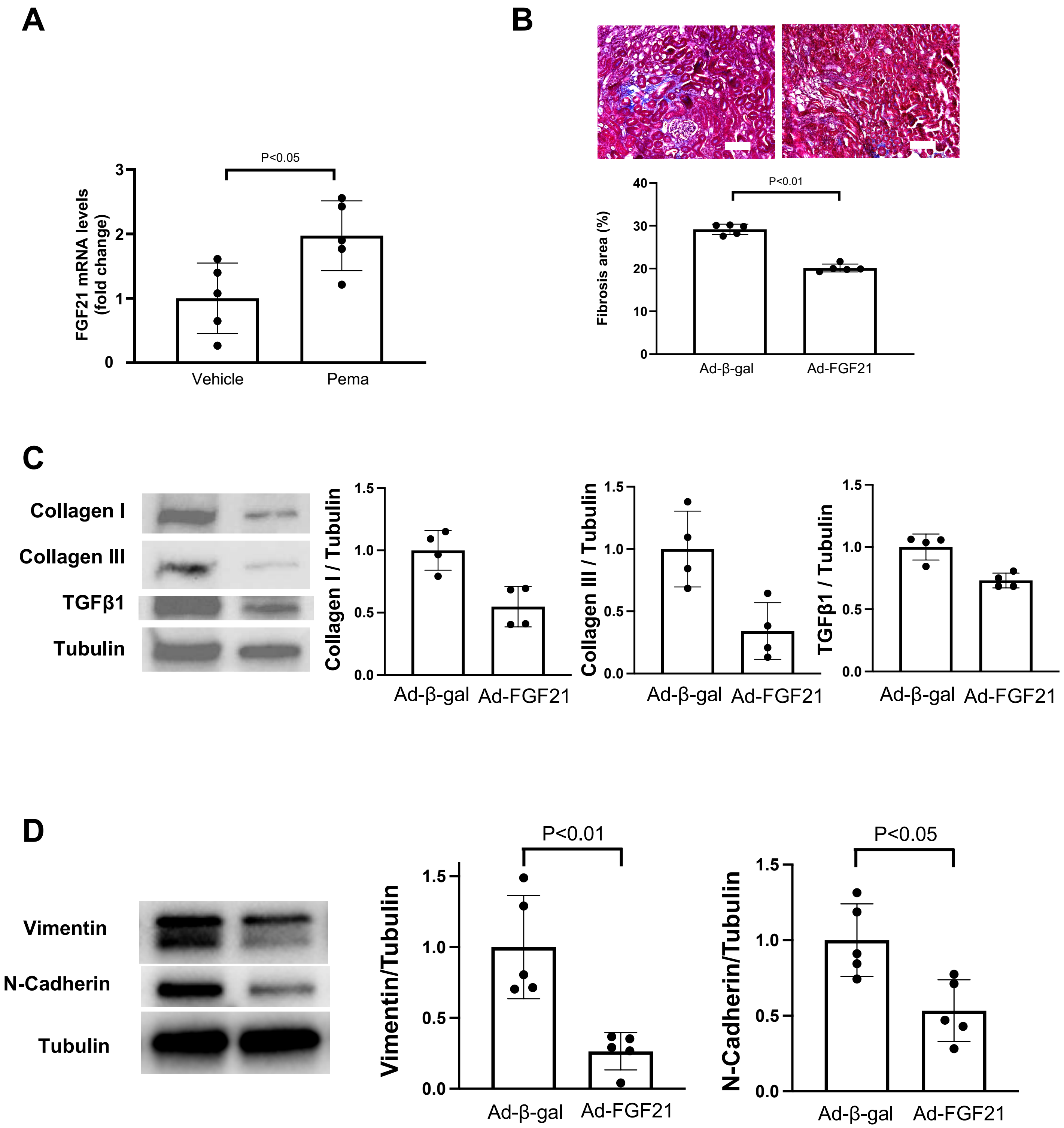
A
P<0.05
FGF21 mRNA levels (fold change)
0
1
2
3
Vehicle
Pema
B
P<0.01
Fibrosis area (%)
0
10
20
30
40
Ad-β-gal
Ad-FGF21
C
Collagen I
Collagen III
TGFβ1
Tubulin
Collagen I / Tubulin
Collagen III / Tubulin
TGFβ1 / Tubulin
0.0
0.5
1.0
1.5
Ad-β-gal
Ad-FGF21
D
Vimentin
N-Cadherin
Tubulin
P<0.01
P<0.05
Vimentin/Tubulin
N-Cadherin/Tubulin
0.0
0.5
1.0
1.5
Ad-β-gal
Ad-FGF21

Figure S3

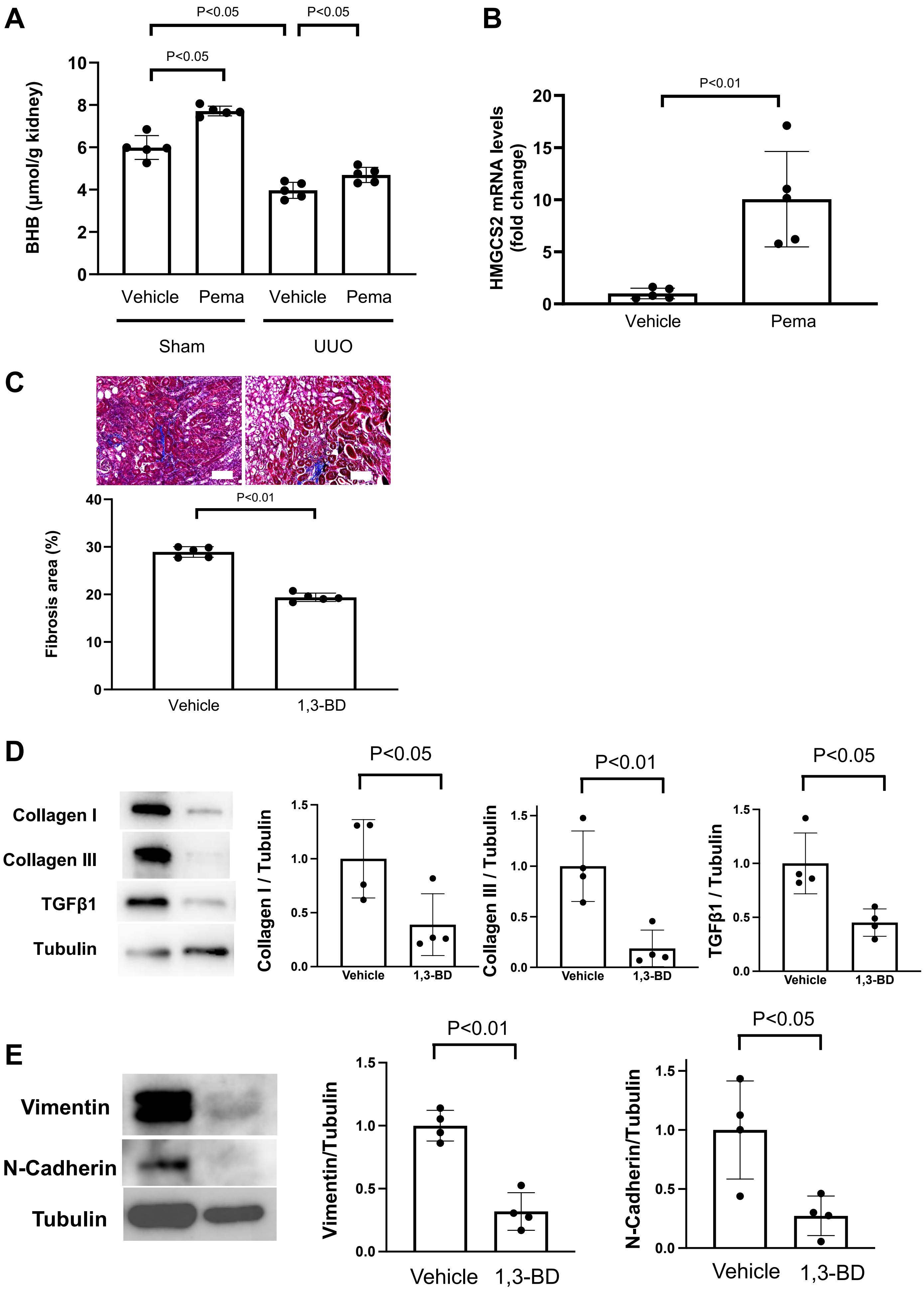
A
P<0.05
P<0.05
P<0.05
BHB (µmol/g kidney)
Vehicle
Pema
Vehicle
Pema
Sham
UUO
B
P<0.01
HMGCS2 mRNA levels (fold change)
Vehicle
Pema
C
P<0.01
Fibrosis area (%)
Vehicle
1,3-BD
D
Collagen I
Collagen III
TGFβ1
Tubulin
P<0.05
Collagen I / Tubulin
P<0.01
Collagen III / Tubulin
P<0.05
TGFβ1 / Tubulin
Vehicle
1,3-BD
E
Vimentin
N-Cadherin
Tubulin
P<0.01
Vimentin/Tubulin
P<0.05
N-Cadherin/Tubulin
Vehicle
1,3-BD

Figure S4

A

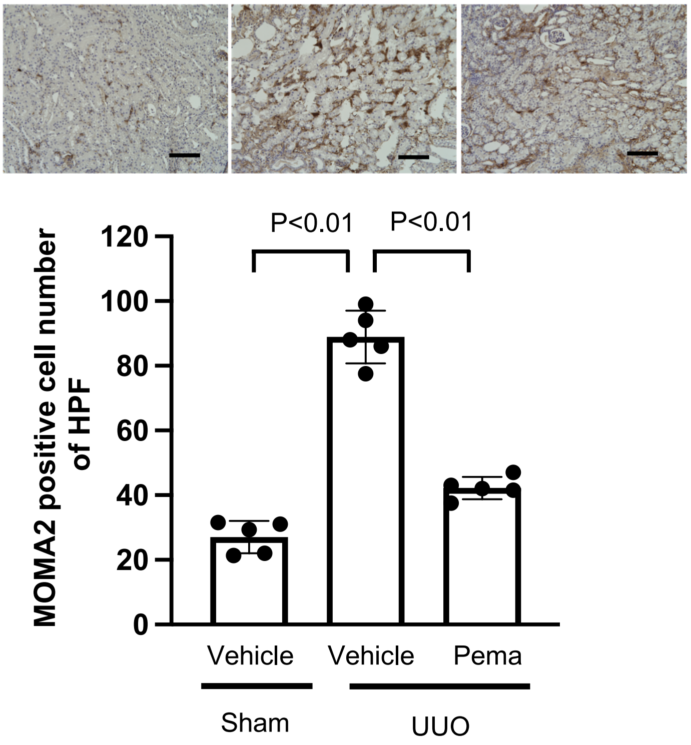

B

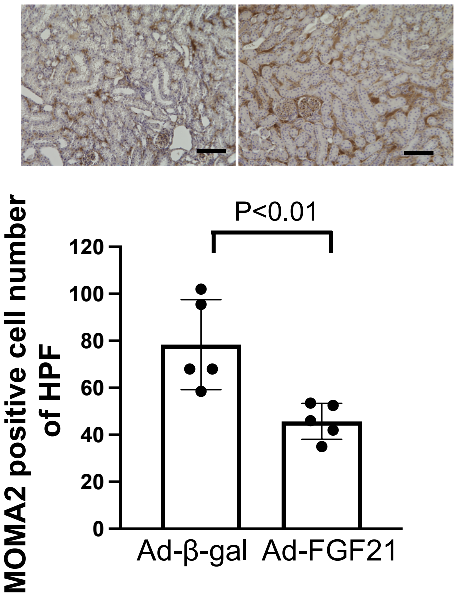

C

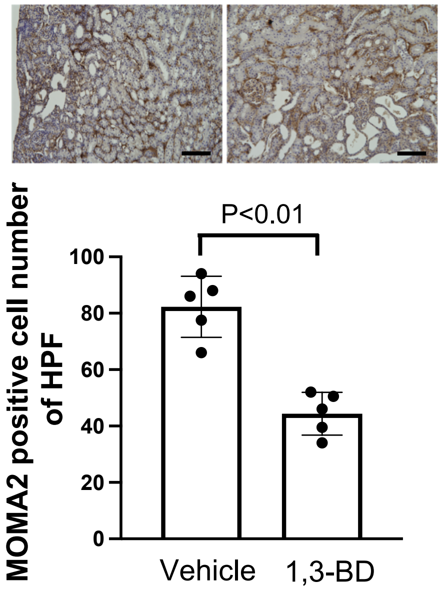

Supplement: Supplementary file 1 — Figures S1–S4. [file PHY2-13-e70135-s001.pdf]
